# Risky sexual behaviors and their association with overweight and obesity among adolescent students: a cross-sectional study

**DOI:** 10.31744/einstein_journal/2019AO4782

**Published:** 2019-06-17

**Authors:** Alaine Souza Lima, Ana Carolina Rodarti Pitangui, Mayra Ruana de Alencar Gomes, Rachel Mola, Rodrigo Cappato de Araújo

**Affiliations:** 1 Universidade de Pernambuco, Petrolina, PE, Brasil.

**Keywords:** Sexual behavior, Adolescent behavior, Overweight, Obesity, Child, Comportamento sexual, Comportamento do adolescente, Sobrepeso, Obesidade, Criança

## Abstract

**Objective:**

To determine the prevalence of risky sexual behavior and its association with overweight and obesity among adolescent students.

**Methods:**

This is a cross-sectional study, conducted in public schools with adolescents aged 12 to 17 years. We used the questionnaire Youth Risk Behavior Survey. The body mass index percentiles were calculated by means of table from the Center for Control and Prevention of Diseases. Possible associations were assessed using the χ^2^ test and binary logistic regression model. The odds ratio and 95%CI were calculated.

**Results:**

We evaluated 1,169 students, and 33.9% reported having had sexual intercourse. Of those, 33% did not use a condom during their last intercourse, and 32.7% had had four or more sexual partners thus far. Regarding nutritional status, 15.3% were overweight or obese. In relation to the non-use of condoms, we observed only an association with the number of lifetime sexual partners (OR: 0.50; 95%CI: 0.28-0.88). The number of lifetime sexual partners was associated with males (OR: 9.17; 95%CI: 4.16-20.22), sexual debut age at 13 years or less (OR: 2.51; 95%CI: 1.23-5.13) and drinking alcohol or using drugs before the last intercourse (OR: 6.16; 95%CI: 2.14-17.73).

**Conclusion:**

Risky sexual behavior rates were high and no association was found between risky sexual behaviors and overweight and obesity.

## Resumo

**Objetivo:**

Determinar a prevalência de comportamentos de risco e sua associação com sobrepeso e obesidade em adolescentes escolares.

**Métodos:**

Trata-se de estudo transversal, realizado em escolas públicas com adolescentes de 12 a 17 anos. Foi utilizado o questionário *Youth Risk Behavior Survey*. Os percentis do índice de massa corporal foram obtidos utilizando as tabelas do *Centers for Disease Control and Prevention*. Possíveis associações foram avaliadas usando o teste do χ^2^ e um modelo de regressão logística binária. Foram calculados os valores de *odds ratio* e o IC95%.

**Resultados:**

Foram avaliados 1.169 estudantes, e 33,9% reportaram ter tido relação sexual. Desse grupo, 33% não utilizaram preservativo na última relação sexual, e 32,7% relataram ter tido quatro ou mais parceiros na vida. Considerando o estado nutricional, 15,3% apresentavam sobrepeso ou obesidade. Em relação ao não uso de preservativo, somente foi observada associação com o número de parceiros sexuais na vida (OR: 0,50; IC95%: 0,28-0,88). O número de parceiros sexuais na vida apresentou associação com o sexo masculino (OR: 9,17; IC95%: 4,16-20,22), idade da primeira relação sexual inferior a 13 anos (OR: 2,51; IC95%: 1,23-5,13) e uso de álcool ou drogas antes da última relação sexual (OR: 6,16; IC95%: 2,14-17,73).

**Conclusão:**

As taxas de comportamento sexual de risco foram altas, e não foi observada sua associação com sobrepeso e obesidade.

## Introduction

Sexual debut among adolescents is occurring earlier and earlier.^(1)^ The impact of the current rates of HIV infections and other sexually transmitted infections (STI) among adolescents is relevant for public health, and poses a threat to the health of young people.^(2)^ Among the factors that may also be considered risky regarding sexual behavior are the age of sexual initiation and the number of sexual partners.^(3)^

Furthermore, obesity may be related to sexual behaviors that increase the risk of acquiring STI.^(2)^ Overweight and obesity can influence the sexual behavior of adolescents, because body mass index (BMI) is associated with psychological factors of physical attractiveness.^(4)^

In order to explain this relation, two theories have been suggested: the first proposes that the association between BMI and sexual risk is mediated by psychosocial risk factors. The psychosocial consequences of obesity, including increased depression and social problems, may affect the ability to have positive loving relationships and negotiate safe sex practices, leading to increased sexual risk.^(5)^ The second theory suggests that overweight and obesity can be seen as factors related to lower sexual risk because they reduce the number of partners and sexual frequency.^(5)^ Another possible mechanism for associations between obesity and risky sexual behavior involves early sexual initiation^(2)^ due to early sexual maturation related to the presence of obesity in childhood.^(6)^

Some studies have found an association between high BMI and risky sexual behaviors, such as not using condoms and consuming alcohol or other drugs before the last intercourse.^(2,7-9)^ In addition, weight misperception has also been identified as an important factor associated with risky behaviors.^(10)^

Due to the increase in obesity rates among adolescents, understanding the relation between BMI and sexual behavior becomes important because certain behaviors established during adolescence may compromise health in the short and long run.^(11,12)^

## Objective

To determine the prevalence of risky sexual behavior and its association with overweight and obesity among adolescent students.

## Methods

This is a cross-sectional epidemiological study, performed in state public schools of Petrolina (PE, Brazil). The study was approved by the Research Ethics Committee of *Universidade de Pernambuco*, CAAE: 24288213.2.0000.5207.

The study population consisted of adolescents enrolled in these schools. The distribution of the sample was done by school size and class period (daytime) to ensure sample proportionality. To support sample planning, schools were classified into three categories: small (less than 200 students); midsize (200 to 499 students); and large (500 students or more).

For sample selection, we used cluster sampling in two stages, with the school and class representing the sampling units in the first and second stages, respectively. All 29 public and urban schools from the city of Petrolina (PE) were considered eligible for the study. Initially, the stratification was carried out for the draw to be divided by school size. After all stages, we reached a number of nine schools, accounting for 31.03% of public schools in the city.

For sample quantification, we considered a population of 25,635 students, 95% confidence interval (95%CI); maximum tolerable error of 5 percentage points; sample loss of 20%; and an estimated prevalence of 50%, because this study covered diverse risky sexual behaviors, with different frequencies of occurrence. We applied a 2.0-point design effect, totaling 948 adolescents. However, to ensure good proportionality of subjects per schools and classes, we chose to assess a minimum of 17 students per class. Thus, the present study evaluated 1,226 adolescents.

The study included adolescents who met the following criteria: age between 12 and 17 years, enrolled in educational institutions located in the urban area of Petrolina (PE). The study excluded adolescents who had physical or psychological diseases or disorders that interfered with data collection; those who handed incomplete forms or forms with mistakes; and those who did not accept having their height and/or body weight measured.

For the socioeconomic assessment, we used a structured questionnaire, and risky behaviors were assessed through the Brazilian version of the Youth Risk Behavior Survey (YRBS).^(13)^ The anthropometric evaluation included the measurement of body weight and height. Both variables were used to calculate BMI, which was categorized as percentiles for each age and sex using the criteria of the Centers for Disease Control and Prevention (CDC).

Before data collection, a pilot study was conducted to test the applicability of the instruments, make corrections, and identify possible biases or limitations in the research procedures. Data were collected in a state public school of Petrolina (PE), with a sample of 80 adolescents. Regarding procedures, the project was presented and then the questionnaires were distributed to the adolescents, who filled them out in the classroom without the teacher’s presence. Upon completion of the questionnaire, the researcher collected the forms and later recorded the anthropometric data.

### Study variables

The dependent variables used in this study were condom use, categorized as “yes” or “no”, and the number of lifetime sexual partners, categorized as “up to three partners” and “four or more partners”.

The independent variables were: socioeconomic data (sex, age, race/ethnicity, religion, marital status, number of children, level of education of parents and monthly family income); sexual behavior variables (had initiated sexual activity, age of sexual initiation, number of sexual partners in the last three months, number of lifetime sexual partners, alcohol consumption or drug use before last sexual intercourse and contraceptive method for the last sexual intercourse); body weight perception; misperception of body weight; intimate partner violence (IPV); and BMI.

For BMI classification, adolescents <5^th^ percentile were considered underweight; ≥5^th^ and <85^th^ percentile, eutrophic; ≥85^th^ and <95^th^ percentile, overweight; and those ≥95^th^ percentile, obese.^(14)^ To verify the misperception of body weight, we compared the categories of BMI to perception of body weight.

Intimate partner violence was assessed by the questions: “During the past 12 months, did you suffer any physical aggression from your partner such as slaps, punches or kicks?” and “Have you ever been forced by your partner to have sex against your will?” Participants who answered “yes” to any of these questions were considered to have a history of IPV.^(10)^

### Data analysis

The data analysis was performed using the software Statistical Package for Social Science (SPSS), version 20.0. The descriptive analysis for categorical variables included distribution of absolute and relative frequencies. For numeric variables, central tendency and dispersion values were calculated according to data distribution, which was verified by Kolmogorov-Smirvov test.

To verify possible factors associated with the dependent variables, a binary logistic regression analysis was performed, using the odds ratio (OR) and their respective 95%CI. A bivariate model of association (crude analysis) was constructed in advance to verify the presence and/or magnitude of the associations between each independent variable and the dependent variable. The model of binary logistic regression adopted was use of the manual backward method. The decision to keep or remove the variables from the model was determined by the analysis of OR values, deviance statistics, changes in the *p* value of the variables, and confidence interval. With this model, we found two competing models and the criterion for choosing the best model was based on the lowest values observed in the Akaike Information Criterion (AIC) and Bayesian Information Criterion (BIC) statistics. Finally, the adjustment quality of the model was determined by the analysis of multicollinearity by the variance inflation factor (VIF), Hosmer-Lemeshow test, and the analysis of influential points. All tests were two-tailed, and all analyses adopted a significance level of p<0.05.

## Results

The study evaluated 1,226 students, but only 1,169 were included in the final data analysis − 57 subjects were excluded due to missing relevant information on their questionnaires. The age range was 12 to 17 years.

Regarding socioeconomic characteristics, we found that 56.6% (n=660; 95%CI: 53.6-59.3) of adolescents were female; 55.0% (n=639; 95%CI: 52.1-57.9) identified themselves as brown; 50.1% (n=575; 95%CI: 47.1-53.0) were catholic; 94.9% (n=1,088; 95%CI: 93.4-96.1) were single; and 66.5% (n=453; 95%CI: 62.8-70.0) had a monthly family income of up to two Brazilian minimum monthly wages. In relation to parents’ education, 33.8% (n=273; 95%CI: 30.7-37.0) reported that their mother had completed junior school or had not finished high school, and 34.8% (n=280; 95%CI: 31.5-38.2) reported that their father had finished junior school or had not finished high school.

In the sample, 33.9% (n=388; 95%CI: 31.2-36.8) reported having had sexual intercourse. Table 1 shows the distribution of the adolescents’ sexual behavior. We found that only 1.2% (n=13; 95%CI: 0.6-2.0) of the students reported having children. Of the individuals evaluated, 3.5% (n=40; 95%CI: 2.4-4.6) reported a history of IPV: 1.5% (n=17; 95%CI: 0.9-2.3) reported being forced to have sexual intercourse and 2.0% (n=23; 95%CI: 1.3-3.0) experienced physical aggression by the partner.

**Table 1:** Sexual behavior among adolescents who reported having initiated sexual activity

Variables	n (%)	95%CI
Sexual initiation age (n=387), years		
≤13	159 (41.1)	36.1-46.2
≥14	228 (58.9)	53.8-63.9
Number of lifetime sexual partners (n=382)		
1	149 (39.0)	34.1-44.1
2-3	108 (28.3)	23.8-33.1
4-5	53 (13.9)	10.6-17.7
≥6	72 (18.8)	15.0-23.1
Number of sexual partners in the last three months (n=378)		
None	107 (28.3)	23.8-33.1
1-5	256 (67.7)	62.7-72.4
≥6	15 (4.0)	2.2-6.5
Alcohol and drug use before the last sexual intercourse (n=376)		
Yes	48 (12.8)	9.6-16.6
No	328 (87.2)	83.4-90.4
Condom use at the last sexual intercourse (n=379)		
Yes	254 (67.0)	62.0-71.7
No	125 (33.0)	28.3-38.0
Contraceptive method at the last sexual intercourse (n=378)		
No method	69 (18.3)	14.5-22.5
Contraceptive pill	43 (11.4)	8.4-15.0
Condom	199 (52.6)	47.5-57.8
Contraceptive pill and condom	27 (7.1)	4.8-10.2
Other methods	40 (10.6)	7.7-14.1

The total number may vary due to missing values. 95%CI: 95% confidence interval.

Regarding height and weight, median, maximum, and minimum were 1.63m (1.36m to 1.95m) and 53.2kg (26.1kg to 106.5kg), respectively. Data about the nutritional status of the adolescents according to BMI and body weight perception are displayed in table 2. The comparison between the BMI categories and perception of body weight showed that 49.4% (n=574; 95%CI: 46.5-52.3) of adolescents had a misperception of body weight; in that, 51.1% (n=336; 95%CI: 47.2-55.0) of females and 47.1% (n=238; 95%CI: 42.7-51.1) of males.

**Table 2:** Body mass index classification and perception of body weight of adolescents (n=1,169)

Variables	Female	Male	Total
	n (%); 95%CI	n (%); 95%CI	n (%); 95%CI
Body mass index			
Low weight	40 (6.1); 4.4-8.2	42 (8.3); 6.0-11.0	82 (7.0); 5.6-8.6
Eutrophic	515 (78.0); 74.7-81.1	393 (77.2); 73.3-80.8	908 (77.7); 75.2-80.0
Overweight	72 (10.9); 8.6-13.5	51 (10.0); 7.5-12.9	123 (10.5); 8.8-12.4
Obesity	33 (5.0); 3.5-6.9	23 (4.5); 2.9-6.7	56 (4.8); 3.6-6.1
Body weight perception			
Very or somewhat lower than expected weight	203 (30.9); 27.4-34.6	187 (37.0); 32.5-41.1	390 (33.6); 30.8-36.4
Expected weight	236 (35.9); 32.2-39.7	213 (42.2); 37.5-46.3	449 (38.6); 35.8-41.5
A little higher than expected weight	168 (25.6); 22.3-29.1	86 (17.0); 13.7-20.4	254 (21.9); 19.5-24.3
Much higher than expected weight	50 (7.6); 5.7-9.9	19 (3.8); 2.3-5.8	69 (5.9); 4.6-7.4

95%CI: 95% confidence interval.

Table 3 presents the results of the independent variables in the final model of logistic regression. In this analysis, only the number of partners showed a significant association with condom use during their last sexual intercourse.

**Table 3:** Binary logistic regression analysis between the non-use of condoms and the independent variables

Independent variables	Non-use of condoms	

	OR (95%CI)	p value
Mother's level of education		
Incomplete junior school or lower	0.44 (0.17-1.13)	0.083
Complete high school or higher	1	
Body mass index		
Low weight	1	
Eutrophic	0.44 (0.17-1.13)	0.087
Overweight and obesity	0.42 (0.12-1.44)	0.166
Body weight perception		
Very or somewhat lower than expected weight	1	
Expected weight	0.75 (0.42-1.35)	0.340
Very or somewhat higher than expected weight	1.35 (0.65-2.82)	0.418
Number of lifetime sexual partners		
≤3	1	
≥4	0.50 (0.28-0.88)	0.016
Intimate partner violence		
Yes	2.26 (0.93-5.48)	0.071
No	1	

p<0.05. OR: odds ratio; 95%CI: 95% confidence interval.

Table 4 shows the results of the regression model, showing that sex, sexual debut age and the use of alcohol or drugs were significantly associated with the number of sexual partners.

**Table 4:** Binary logistic regression analysis between the number of partners and the independent variables

Independent variables	≥4 partners	

	OR (95%CI)	p value
Age, years		
≤14	1	
≥15	2.35 (0.84-6.55)	0.104
Sex		
Female	1	
Male	9.17 (4.16-20.22)	<0.001
Family income, minimum wage		
Up to 2	0.80 (0.40-1.62)	0.534
>2	1	
Body mass index		
Low weight	1	
Eutrophic	2.10 (0.58-7.56)	0.255
Overweight and obesity	0.94 (0.19-4.53)	0.936
Sexual initiation age, years		
≤13	2.51 (1.23-5.13)	0.011
≥14	1	
Alcohol and drug use before the last sexual intercourse		
Yes	6.16 (2.14-17.73)	0.001
No	1	
Intimate partner violence		
Yes	2.80 (0.70-11.14)	0.145
No	1	

p<0.05. OR: odds ratio; 95%CI: 95% confidence interval.

## Discussion

Regarding sexual behavior of the adolescents evaluated, the majority reported having had only one partner, followed by 32.7% of adolescents who had four or more partners. Corroborating these findings, Lowry et al.,^(2)^ found a similar prevalence of young people who had four or more partners, which can be explained by the fact that adolescence is a time for experimenting during which it is common to change partners. However, the high number of partners is concerning health wise, as it can increase the risk of transmission of HIV and other STI.^(12)^

Most teenagers used a condom during the last intercourse. Nevertheless, it is important to note that about one third of the young people who had begun their sexual life did not use condoms during their last intercourse. This shows that, although young people have access to information about STI, that is not enough to establish preventive actions.

Regarding the adolescents’ nutritional status according to BMI, the prevalence of overweight and obesity was 10.5% and 4.8%, respectively, and was higher among girls. These numbers were lower than those found in other national and international studies.^(2,15,16)^

In addition to the BMI analysis, another important variable that can influence behavior is the perception of body weight. In this study, most students reported being within their expected weight. However, we observed that the prevalence of individuals who considered themselves to be underweight was higher among men. On the other hand, the prevalence of students who thought they were above their ideal weight or very overweight was higher among women. Therefore, these findings can be explained by the tendency of girls to overestimate and of boys to underestimate their body weight.

In the analysis of the association between independent variables and the non-use of condoms, we found that the variables that were likely to be included in the regression model were the mother’s level of education, BMI, body weight perception, number of lifetime sexual partners, and IPV. However, after the binary logistic regression analysis, the only variable that remained associated was the number of sexual partners in life. The results showed that individuals who reported having had four or more sexual partners had 50% less chances of not using a condom during their last sexual intercourse. From this data, we can infer that adolescents who had more partners used condoms more often. However, this study did not assess the type of relationship as stable or casual - a higher number of partners is associated with casual relationships.

Calazans et al.,^(17)^ found significant differences in condom use among young people according to the type of relationship during the last intercourse − condom use with casual partners occurs more frequently than with steady partners. Thus, adolescents believe that stable relationships have a lower risk of infections, resulting in a bigger challenge when it comes to condom use.

In the association between independent variables and the number of lifetime sexual partners, the variables included in the regression model were age, sex, family income, BMI, age of sexual initiation, alcohol consumption or drug use before the last intercourse, and IPV. However, after the binary logistic regression, the variables that remained were related to sex, age of sexual initiation and intake of alcohol or drugs before the last sexual intercourse.

Our results found that male adolescents were 9.17 times more likely to have four or more sexual partners in their lifetime. However, this difference between sexes was expected, since sexuality is strongly influenced by the asymmetrical relations between sexes observed in society. For boys, sexual initiation and changing partners are considered as more natural, unlike what is expected from girls, who are supposed to display a more chaste behavior.

Regarding the age of sexual initiation, students who started sexual activity at the age of 13 years or less had 2.51 times more chance of having four or more lifetime sexual partners. However, this relation was already expected because early sexual initiation shows an association with a higher number of sexual partners throughout life. According to the literature, the association between an early initiation of sexual activity and STI is mediated by the number of sexual partners.^(18)^

The consumption of alcohol or drugs before the last sexual intercourse was presented as a risk factor that increases by 6.16 times the chances of having four or more lifetime sexual partners; when young people are under the influence of these substances, they are more likely to have unplanned sex during a chance encounter.^(19)^

The BMI variable was included in the regression models with the dependent variables no condom use and number of sexual partners. However, the regression analysis did not remain associated with these variables, which is in line with the results of other studies that also found no association between BMI and risky sexual behavior.^(10,20)^ On the other hand, other authors found an association between high BMI and risky sexual behaviors, such as not using condoms and consumption of alcohol or drugs before their last intercourse.^(2,7-9)^

Differently, Averett et al.,^(21)^ found that overweight girls have a lower probability of having vaginal intercourse without a condom, and Nagelkerke et al.,^(4)^ found that overweight adults reported fewer sexual partners in the last year than their eutrophic peers. Therefore, no consensus is found in the literature on the theme and the results vary greatly. That may be due to the different methodologies employed regarding the characterization of the samples, with different profiles and sizes and different BMI cutoff points for the evaluation of nutritional status.

Regarding our results, which showed no association between BMI and sexual behavior, we highlight the fact that all other studies were conducted in the United States. It is important to point out that cultural characteristics can influence the behavior of adolescents. Furthermore, in this study, the prevalence of overweight and obesity was low, which may have influenced the results.

Although the perception of body weight was included only in the model with the dependent variable “non-use of condoms”, after the regression analysis, the association between these factors disappeared. That may contradict the findings by Akers et al.,^(10)^ which revealed that adolescents who perceived themselves overweight were less likely to report condom use during their last intercourse. However, their study evaluated only female individuals.

This research provides relevant information about adolescents in this region of Brazil and can assist in decisions regarding health campaigns for this population. Nevertheless, we highlight some limitations related to our study. The data was obtained in a small town in the state of Pernambuco; hence, their local, social and cultural reality may not reflect the profile of the entire country. The cross-sectional design does not allow the analysis of the causal effect of these behaviors. Moreover, the questionnaire was done by self-report, which could result in increased susceptibility to recall bias, which is an intrinsic limitation of retrospective and cross-sectional studies.^(22)^ Finally, all participants came from public schools, which limits result reproducibility to private sector students.

We suggest further studies on the subject with a longitudinal design, analyzing adolescents from public and private educational institutions from different regions.

## Conclusion

The risky sexual behavior rates were high, and there was no association between overweight and obesity and the risky sexual behaviors analyzed.
